# Residual Endotoxin Contaminations in Recombinant Proteins Are Sufficient to Activate Human CD1c^+^ Dendritic Cells

**DOI:** 10.1371/journal.pone.0113840

**Published:** 2014-12-05

**Authors:** Harald Schwarz, Maria Schmittner, Albert Duschl, Jutta Horejs-Hoeck

**Affiliations:** Department of Molecular Biology, University of Salzburg, Salzburg, Austria; Virginia Polytechnic Institute and State University, United States of America

## Abstract

Many commercially available recombinant proteins are produced in *Escherichia coli*, and most suppliers guarantee contamination levels of less than 1 endotoxin unit (EU). When we analysed commercially available proteins for their endotoxin content, we found contamination levels in the same range as generally stated in the data sheets, but also some that were higher. To analyse whether these low levels of contamination have an effect on immune cells, we stimulated the monocytic cell line THP-1, primary human monocytes, *in vitro* differentiated human monocyte-derived dendritic cells, and primary human CD1c^+^ dendritic cells (DCs) with very low concentrations of lipopolysaccharide (LPS; ranging from 0.002–2 ng/ml). We show that CD1c^+^ DCs especially can be activated by minimal amounts of LPS, equivalent to the levels of endotoxin contamination we detected in some commercially available proteins. Notably, the enhanced endotoxin sensitivity of CD1c^+^ DCs was closely correlated with high CD14 expression levels observed in CD1c^+^ DCs that had been maintained in cell culture medium for 24 hours. When working with cells that are particularly sensitive to LPS, even low endotoxin contamination may generate erroneous data. We therefore recommend that recombinant proteins be thoroughly screened for endotoxin contamination using the limulus amebocyte lysate test, fluorescence-based assays, or a luciferase based NF-κB reporter assay involving highly LPS-sensitive cells overexpressing TLR4, MD-2 and CD14.

## Introduction

Many commercially available recombinant proteins, especially small and non-glycosylated proteins, are produced in *Escherichia coli (E.coli)*. Although this expression system has many advantages, including rapid expression, high yields, ease of culture and low cost [1], the proteins recovered may be contaminated by endotoxin. This highly complex lipopolysaccharide (LPS) is a major component of the outer membrane of most gram-negative bacteria and is regarded as the main virulence factor of the latter [2]. LPS is recognized by a receptor complex composed of TLR4, CD14 and MD-2 [3]. Upon recognition and binding of microbial ligands by the extracellular domains of this receptor complex, the intracellular portion recruits adaptor kinases which enable signal transduction, most likely through activation of the transcription factor nuclear factor-kappa B (NF-κB) [4], [5]. In human monocytes and macrophages these transcriptional responses culminate in the release of pro-inflammatory cytokines, including TNFα, IL-1β, IL-6, IL-8, and IL-12 [6], [7].

In data sheets accompanying commercially produced recombinant proteins, the amount of bacterial contamination is usually stated in endotoxin units (EU), and most suppliers guarantee contamination levels of less than 1 EU, which is approximately equivalent to 100 pg of *E. coli* LPS per microgram of recombinant protein [8]. Based on that level, protein preparations at concentrations ranging from 10–1000 ng/ml may be contaminated with 1-100 pg LPS. Because the vast majority of *in vitro* studies have reported on endotoxin effects induced by concentrations between 1 and 100 ng/ml, the current study investigates the effects of very low endotoxin concentrations ranging from 0.002–2 ng/ml on human immune cells, as these concentrations are equivalent to the amount of residual contamination present in recombinant proteins.

## Materials and Methods

All studies involving human cells were conducted in accordance with the guidelines of the World Medical Association's Declaration of Helsinki.

### Isolation and cultivation of cells and cell lines

THP-1 cells were cultivated in RPMI 1640 medium (Sigma-Aldrich, Vienna, Austria) supplemented with 10% heat-inactivated (i.a.) fetal bovine serum (FBS; PAA, GE Healthcare, Pasching, Austria), 100 U/ml penicillin (PAA), 100 µg/ml streptomycin (PAA) and 2 mM L-glutamine (Gibco, Life Technologies, Lofer, Austria).

Monocytes and moDCs were generated from buffy coats from healthy, anonymous donors (provided by the blood bank Salzburg, Austria) using the adherence method as described before [9]. Briefly, peripheral blood mononuclear cells (PBMCs) were isolated from buffy coats by gradient centrifugation using Ficoll-Paque PLUS (PAA, GE Healthcare, Pasching, Austria). After erythrocyte lysis using ACK buffer (150 mM ammonium chloride, 10 mM potassium bicarbonate, 0.1 mM EDTA) and extensive washing with RPMI 1640 medium, cells were left to adhere for 90 min at 37°C and 5% CO_2_ in six-well plates in RPMI 1640 medium containing 10% i.a. FBS, 100 U/ml penicillin, 100 µg/ml streptomycin, 2 mM L-glutamine and 50 µM 2-mercaptoethanol. Non-adherent cells were then removed by extensive washing using warm RPMI 1640 medium. For the generation of moDCs, adherent monocytes were stimulated with 50 ng/ml GM-CSF and 50 ng/ml IL-4 (kind gifts from Novartis, Vienna, Austria) for six days. At day 3, 1 vol of the supplemented medium containing fresh cytokines was added.

Primary human CD1c^+^ DCs were isolated via magnetic cell sorting using the Miltenyi CD1c (BCDA-1) + Dendritic Cell Isolation Kit according to the manufacturer's instructions. CD1c^+^ DCs were cultivated in DC-medium (RPMI 1640 medium supplemented with 10% i.a. FBS, 100 U/ml penicillin, 100 µg/ml streptomycin and 2 mM L-glutamine). The purity of monocytes, moDCs and CD1c^+^ DCs was routinely analysed by flow cytometry.

### Reagents and recombinant proteins

*E. coli* LPS 055:B5 was obtained from Sigma–Aldrich, Vienna, Austria. All proteins tested in this study are recombinant human cytokines and were obtained from three different suppliers, labelled supplier 1, 2 and 3. According to the manufacturers' data sheets, these recombinant proteins were routinely tested for endotoxin contamination by unspecified LAL tests. However, we do not disclose the names of the manufacturers or products in this study due to the proprietary nature of this information.

### EndoZyme and EndoLISA

The EndoZyme and EndoLISA endotoxin detection assays were purchased from Hyglos GmbH, Bernried am Starnberger See, Germany and performed according to the manufacturer's instructions. Fluorescence was measured using a Tecan Infinite 200 Pro microplate reader. The sensitivity setting (gain) of the fluorescence reader was adjusted by performing the assays one time at automatically detected optimal gain at the 90 min timepoint. This gain setting was then used throughout all further experiments. Standard curves were calculated using a non-linear regression model.

### Transfection of HEK293 cells and luciferase assay

1.2×10^5^ HEK293 cells per well in 500 µl antibiotics-free DMEM medium (Sigma-Aldrich; supplemented with 10% i.a. FBS, 2 mM L-glutamine and 2 mM MEM non-essential amino acids) were plated in 24-well plates. After 24 h, cells were transfected using Lipofectamine 2000 reagent (Invitrogen, Life Technologies, Lofer, Austria) according to the manufacturer's instructions. Briefly, per well, 500 ng DNA and 1.5 µl of Lipofectamine 2000 reagent were diluted in 25 µl of Opti-MEM (Gibco). As DNA, an NF-κB Luciferase reporter (kindly provided by Min Li-Weber [10] and cloned into pGL3Neo in our laboratory) and a TLR4-CD14-MD2 mix was used in a ratio of 4∶1. The mix consists of expression plasmids for TLR4 (in pCDNA3), CD14 (in pCDNA3) and MD-2 (in pEFBOS) (kind gifts from Andrei Medvedev and Douglas Golenbock) in a ratio of 3∶1∶1. Both reactions were mixed, pooled and incubated for 5 min at room temperature (RT). The transfection mix was then added to the wells. After 24 h, the medium was replaced with fresh medium (DMEM supplemented with 10% i.a. FBS, 2 mM L-glutamine, 100 U/ml penicillin, 100 µg/ml streptomycin and 1x MEM (2 mM) non-essential amino acids). Cells were then induced with the appropriate stimuli. After 20 h of induction, supernatants were discarded and 100 µl of lysis buffer (100 mM potassium phosphate, 0.1% Triton X-100, 1 mM DTT) was added to each well. The lysates were transferred to white polystyrene flat-bottom microtitre plates in duplicates. Luciferase activity was measured in a Tecan Infinite 200 Pro microplate reader, where 50 µl of luciferase substrate was added using an automated dispenser.

### Limulus amebocyte lysate (LAL) assay

The Pyrotell-T (Cape Cod, MA, USA) LAL-assay was used according to the manufacturer's instructions. Turbidity was measured every 10 min for at least 1.5 h at 37°C in a Tecan Infinite 200 Pro microplate reader at 405 nm. The time point with the highest resolution between the endotoxin standard samples was chosen for analysis.

### Enzyme-linked immunosorbent assay (ELISA)

The human IL-1β DuoSet ELISA kit was purchased from R&D Systems, Biomedica, Vienna, Austria. All other ELISA kits were purchased from Peprotech, Vienna, Austria. The assays were performed according to the manufacturers' instructions. Briefly, NUNC MaxiSorp flat-bottom 96-well plates (eBioscience, Affymetrix, Vienna, Austria) were coated with a capture antibody overnight and blocked with PBS containing 1% bovine serum albumin (BSA) for 1 h. After washing, cell supernatants were added and incubated for at least 2 h at room temperature (RT). Plates were washed again and the biotinylated detection antibody was added. After incubation for 1 h at RT, plates were again washed and avidin-conjugated horseradish peroxidase was added. After 30 min of incubation and another washing step, the substrate (TMB from Sigma-Aldrich) was added. The reaction was stopped by adding 2 M sulphuric acid. Colour intensity was measured at 450 nm and a reference wavelength of 650 nm was subtracted. The respective standards were used to calculate total protein concentrations.

### Quantitative real-time PCR (qRT-PCR)

Total RNA from cells was isolated using TRI reagent (Sigma-Aldrich) and reverse transcribed with RevertAid H Minus reverse transcriptase (Thermo Scientific, Vienna, Austria) according to the manufacturer's instructions. Quantitative real-time RT-PCR (qRT-PCR) was performed on a Rotorgene 3000 (Corbett Research, Mortlake, Australia) using iQ SYBR Green Supermix (Bio-Rad, Vienna, Austria) and the primers listed below. The large ribosomal protein P0 (RPLP0) was used as a reference. Specificity was controlled by recording a melting curve for the PCR products. Relative mRNA expression levels were calculated using the formula x = 2^−ΔCt^, where Ct represents the threshold cycle of a given gene and ΔCt signifies the difference between the Ct values of the gene in question and the Ct value of the reference gene.

Primer sequences:

Human IL-1β sense 5′-GTA CCT GAG CTC GCC AGT GA-3′ and anti-sense 5′-TCG GAG ATT CGT AGC TGG ATG-3′.

Human RPLP0 sense 5′-GGC ACC ATT GAA ATC CTG AGT GAT GTG-3′ and anti-sense 5′-TTG CGG ACA CCC TCC AGG AAG-3′.

### Flow cytometry

For flow cytometry, 1×10^5^ cells were suspended in 50 µl of ice-cold FACS buffer (PBS supplemented with 3% i.a. FBS) including appropriate amounts of fluorescent-labelled antibodies. As a control for nonspecific binding, one aliquot of cells was labelled with isotype-specific control antibodies in excess concentration. Cells were analysed using a FACS Canto II flow cytometer (BD Biosciences). Primary monocytes, moDCs and CD1c^+^ DCs were gated according to cell-specific surface markers (CD14, CD1a and CD1c, respectively). For analysis of CD40, CD80, CD83 and CD86 surface expression, the median fluorescence intensity (MFI) of 1×10^4^ cells was recorded for every sample. The fold-changes of the MFIs were then calculated compared to the control cells (PBS/BSA-induced cells) of each cell type, to allow direct comparison of the different cell types measured with different voltages and/or compensation settings. For analysis of TLR4 and CD14 surface expression, cells were gated according to the individual isotype controls, and the percentages of TLR4 and CD14 positive cells were used as readout. For statistical analysis, an ANOVA with a Dunnett post-test was performed for each cell type individually, using the MFI values (not the fold-change values), or the percentages of positive cells. Anti-human CD1c-FITC and mouse IgG1-PE isotype control were obtained from BioLegend, Vienna, Austria. Anti-human CD1a-FITC, CD14-FITC, CD14-PE, CD19-PE, mouse IgG1-FITC, mouse IgG2a-APC and mouse IgG2b-PE isotype controls were purchased from Immunotools, Vienna, Austria. Anti-human CD14-APC, CD1a-APC, mouse IgG1-FITC isotype control, CD40-APC, CD80-PE, CD83-APC and CD86-PE were acquired from BD Biosciences, Erembodegem, Belgium. Anti-human CD1c-APC and mouse IgG1-APC isotype control were purchased from R&D Systems, and anti-human TLR4 (CD284)-Alexa Fluor 488 was obtained from eBioscience.

## Results

**Figure 1:**
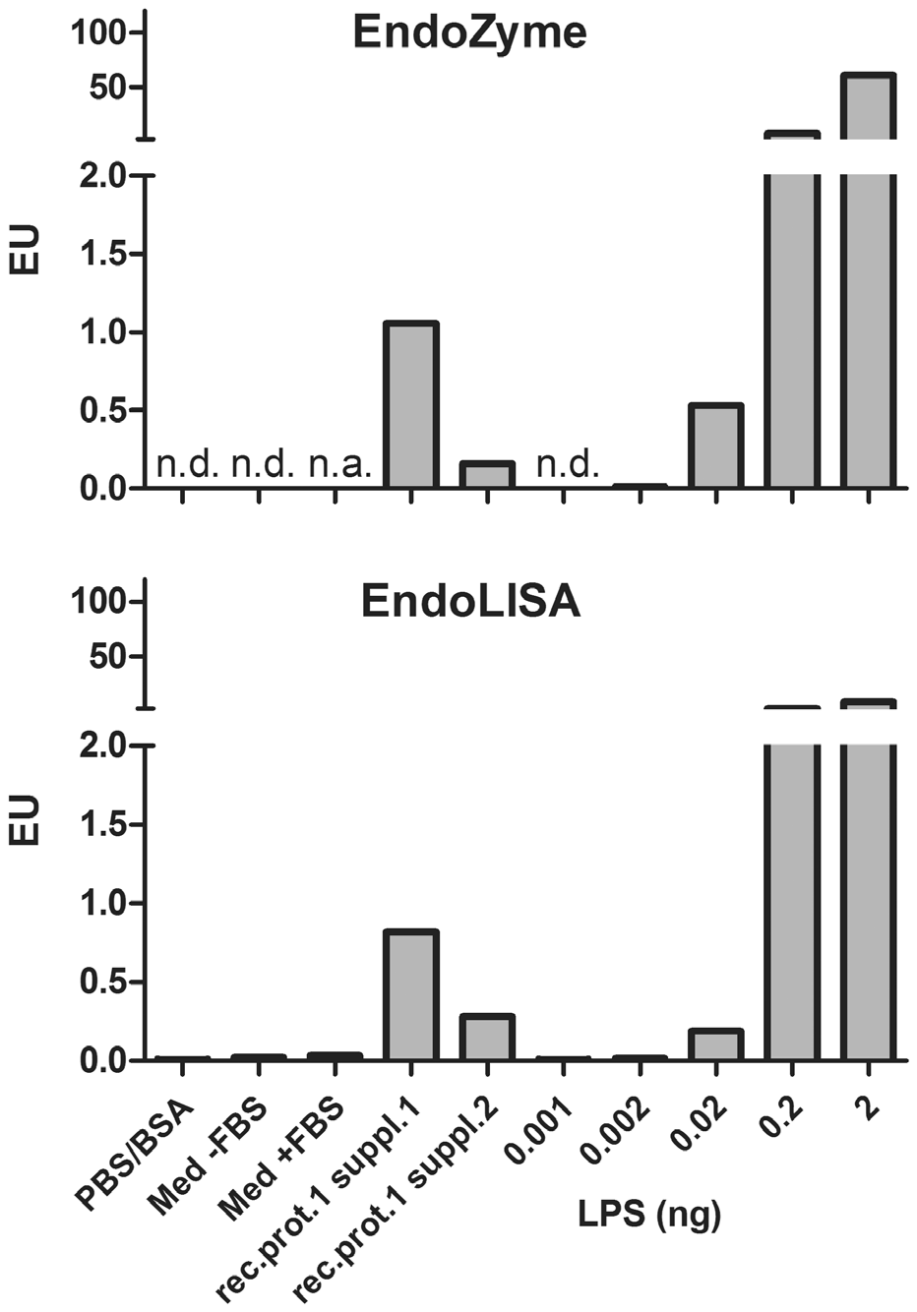
Analysis of endotoxin contents by two commercially available endotoxin detection assays. Endotoxin contamination levels of recombinant protein 1 from suppliers 1 and 2 were measured by EndoZyme and EndoLISA. Results show impurities in 1 µg of recombinant protein 1 from two different suppliers (rec.prot.1 suppl.1 and rec.prot.1 suppl.2). As controls, total amounts of 0.001–2 ng *E.coli* LPS, as well as solvent (PBS containing 0.1% BSA, undiluted), DC-medium without FBS (Med –FBS) and with FBS (Med + FBS; both diluted 1∶10 in endotoxin-free water) were used. Results show mean values of technical duplicates. n.d., not detectable; n.a., not applicable.

To assess whether LPS levels in the range of 0.1–1 EU are actually present in commercial recombinant proteins, we performed LAL assays on five commercially obtained recombinant proteins. As shown in **Table 1**, three proteins had a slightly higher endotoxin amount than stated in the data sheet. Of note, the measured endotoxin levels in recombinant protein 1, a product that was obtained from two different suppliers, were vastly different. Whereas the protein from supplier 2 had barely measurable endotoxin impurities (2 pg/µg protein), the product from supplier 1 was contaminated with 140 pg of endotoxin per microgram of protein, somewhat higher than the maximum specified by the manufacturer (<1 EU or <100 pg). To confirm these endotoxin contaminations, recombinant protein 1 from both suppliers was once more tested by alternative endotoxin detection assays (EndoZyme and EndoLISA) along with increasing concentrations of LPS (**Figure 1**). The two additional assays revealed detectable endotoxin contaminations for protein 1 from supplier 1, although the results provided by the three different assays (LAL, EndoZyme and EndoLISA) were not completely identical and ranged between 0.8 and 1.4 EU.

**Table 1:** Measured impurities in protein preparations from different suppliers evaluated by LAL tests.

	Impurities according to datasheet	Impurities according to LAL Test	Impurities at 100 ng protein
PBS/0.1% BSA	-	**<0.1 EU**	-
DC Medium without FBS	-	**<0.1 EU**	-
DC Medium with 10% i.a. FBS	-	**<0.1 EU**	-
Recombinant protein 1, supplier 1	<1 EU (<0.1 ng/µg protein)	**1.4 EU (0.14 ng/µg protein)**	0.014 ng
Recombinant protein 1, supplier 2	<1 EU (<0.1 ng/µg protein)	**<0.1 EU (0.01 ng/µg protein)**	<0.001 ng
Recombinant protein 2, supplier 1	<0.1 EU (<0.01 ng/µg protein)	**0.32 EU (0.032 ng/µg protein)**	0.003 ng
Recombinant protein 3, supplier 1	<0.1 EU (<0.01 ng/µg protein)	**0.25 EU (0.025 ng/µg protein)**	0.003 ng
Recombinant protein 4, supplier 3	<1 EU (<0.1 ng/µg protein)	**<0.1 EU (0.01 ng/µg protein)**	<0.001 ng

**Figure 2:**
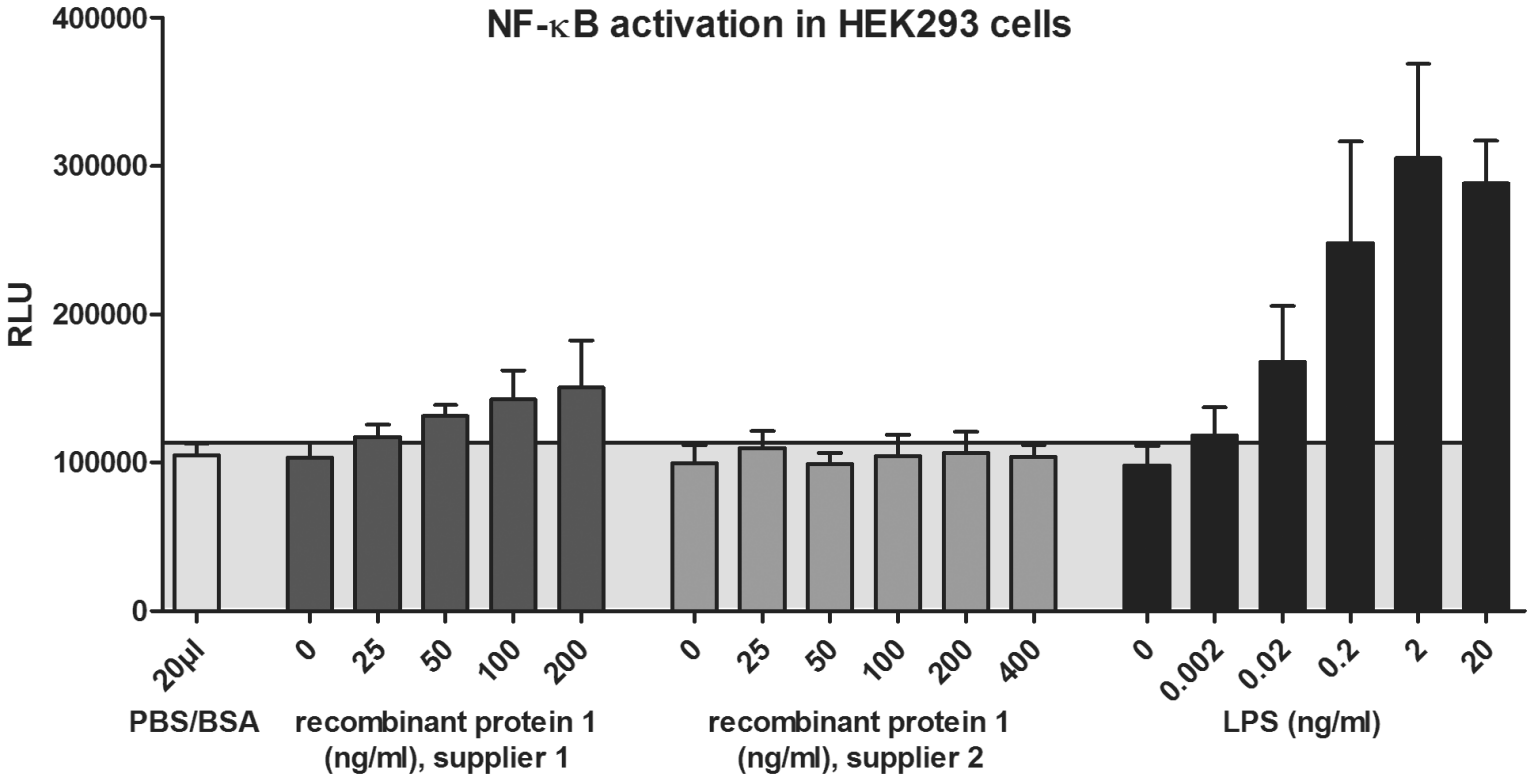
Activation of NF-κB by endotoxin impurities in commercial preparations of a recombinant protein. NF-κB activation in HEK293 cells transfected with an NF-κB luciferase reporter plasmid and plasmids encoding LPS-receptor components (TLR4, CD14, MD-2) is shown. Cells were exposed to recombinant protein 1 from supplier 1 or 2, LPS, or solvent, in the amounts stated. 20 h after induction, luciferase activity was measured. Results show mean and standard deviations of four independent experiments.

To assesses whether these small amounts of LPS are capable of activating NF-κB-signalling, we generated a highly LPS-responsive cell system modified from Peters and colleagues [11] by co-transfecting HEK293 cells with expression plasmids encoding the LPS receptor subunits TLR4, CD14 and MD-2 along with an NF-κB luciferase reporter plasmid. These cells were exposed to different concentrations of recombinant protein 1 from suppliers 1 and 2, as well as to different amounts of LPS. As shown in **Figure 2**, the recombinant protein from supplier 1 induced an increase in NF-κB activity, whereas the protein from supplier 2 did not activate NF-κB (even at 400 ng/ml, twice as high as the maximum protein concentration tested from supplier 1). Interestingly, the protein from supplier 1 induced NF-κB activation in the same range as 0.02 ng/ml LPS. Of note, this LPS concentration is approximately equivalent to the amount of contamination in 100 ng of recombinant protein, as measured in the LAL test above. This experiment clearly shows that even the small amounts of endotoxin contamination found in commercially available recombinant proteins are sufficient to activate NF-κB in a highly sensitive cell system.

**Figure 3:**
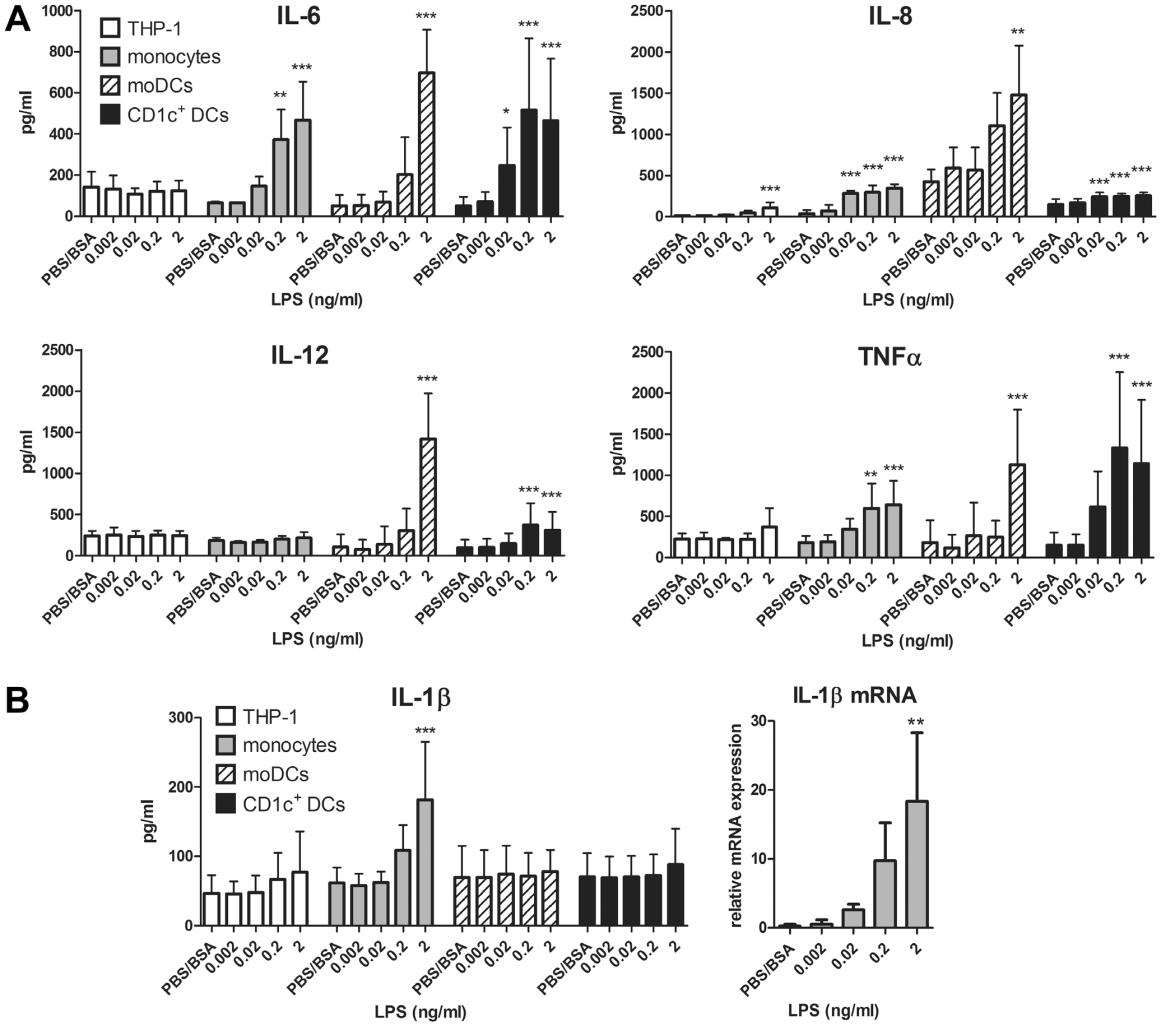
Effects of low LPS concentrations on cytokine production in different human immune cells. **A** 1×10^5^ THP-1 cells, primary human monocytes, monocyte-derived DCs (moDCs), or CD1c^+^ DCs were either stimulated with different concentrations of LPS or solvent (PBS/BSA) as a control. After 16 h, supernatants were harvested and analysed by ELISA. Results show means and SD of at least four independent experiments per cell type. **B** Left panel: Cells were plated and stimulated as described above. After 24 h, supernatants were harvested and analysed for IL-1β expression by ELISA. Results show means and SD of at least five independent experiments per cell type. Right panel: 1×10^5^ monocytes were stimulated as described above. After 6 h, cells were harvested and mRNA levels were measured by qRT-PCR. Results show means and SD of three independent experiments. One-way ANOVA with a Dunnett post-test was performed for each cell type individually. * p<0.05, ** p<0.01, *** p<0.001 compared to the solvent control of the respective cell type.

Because transfected HEK293 cells are a rather artificial cell model, we tested different human immune cells that are naturally sensitive to endotoxins: we stimulated the monocytic cell line THP-1, primary monocytes, *in vitro* differentiated monocyte-derived dendritic cells (moDCs), and primary CD1c^+^ dendritic cells (CD1c^+^ DCs) with very low LPS concentrations. As shown in **Figure 3A**, even 0.02 ng/ml LPS was able to significantly stimulate the production of cytokines. However, we observed some vast differences between the individual cell types in terms of both the types of cytokines they produced and their sensitivity to LPS. THP-1 cells produced only minuscule amounts of IL-8 upon stimulation with the highest LPS-concentration used (2 ng/ml), whereas moDCs secreted considerable amounts of IL-6, IL-8, TNF-α and IL-12 at the same high LPS concentration. In contrast, human monocytes and CD1c^+^ DCs were much more sensitive to LPS, as 0.2 ng/ml LPS was sufficient to induce the release of all tested cytokines, except for IL-12 in monocytes. One important observation in this context is that, in both monocytes and CD1c^+^ DCs, statistically significant amounts of IL-8 were measured in response to 0.02 ng/ml LPS. Moreover, CD1c^+^ DCs additionally produced IL-6 upon activation under these conditions. In contrast, significant release of IL-1β was observed only from monocytes treated with 2 ng/ml LPS, whereas lower LPS concentrations had no significant effect, and none of the other cells types secreted IL-1β in our experimental setup (**Figure 3B, left panel**). IL-1β is expressed as a biologically inactive 31-kDa pro-form that requires post-translational processing by caspase-1 to generate the biologically active cytokine IL-1β [12], [13]. To investigate whether low LPS concentrations would stimulate the post-translational processing of pro-IL-1β or rather induce IL-1β expression at the transcriptional level, we analysed IL-1β mRNA after 6 hours of LPS stimulation (**Figure 3, B right panel**). As IL-1β mRNA expression directly correlates with IL-1β secretion, we assume that low LPS concentrations regulate IL-1β secretion at the transcriptional rather than the post-translational level.

**Figure 4:**
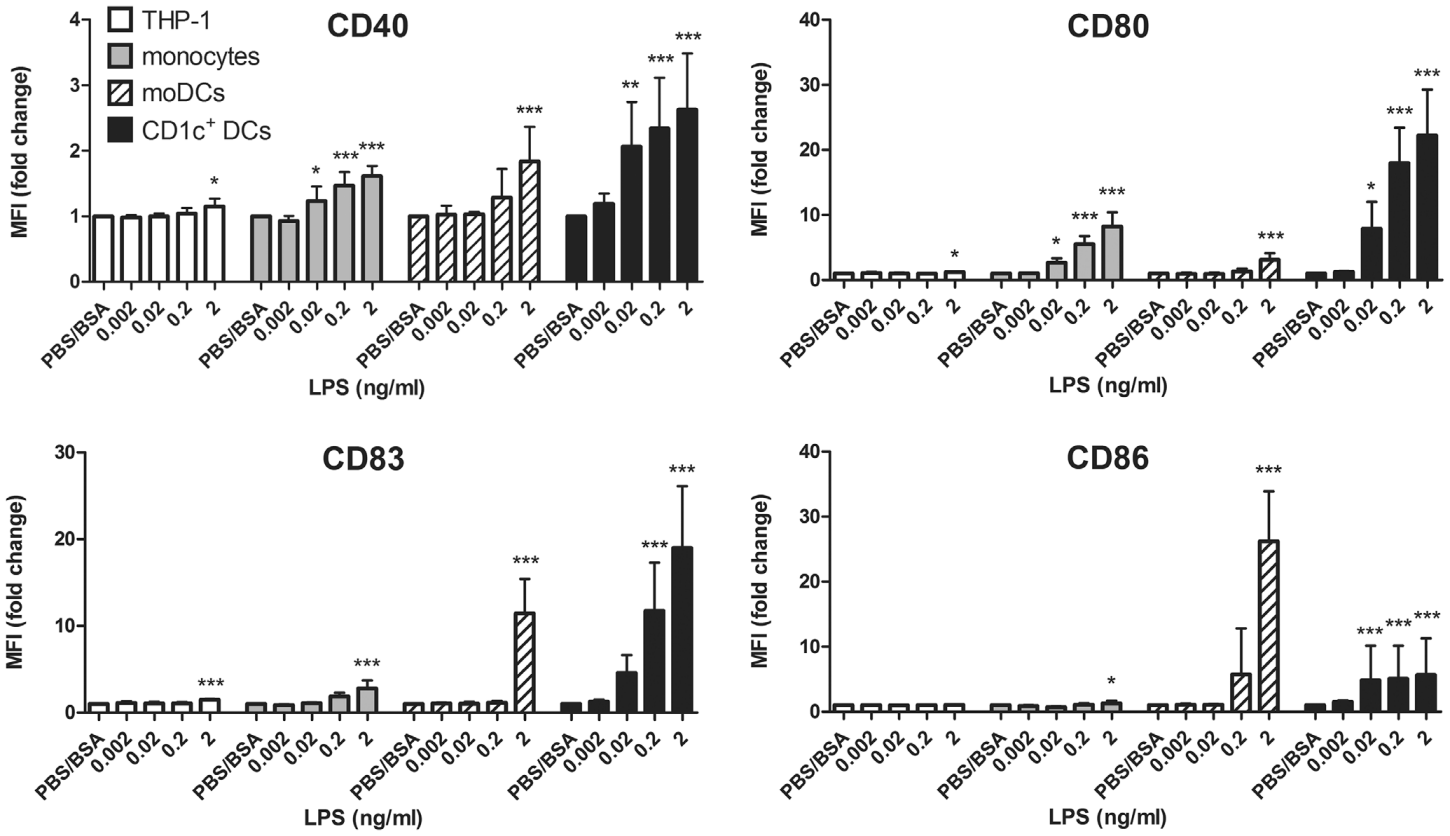
Surface marker expression upon stimulation with low LPS concentrations. Cells were plated and induced as described in Figure 3. After 16 h of induction, cells were harvested and stained for the expression of surface markers using appropriate amounts of fluorescent-labelled antibodies. The median of the fluorescence intensities of 1×10^4^ cells each was recorded by flow cytometry. Fold change values were calculated to compare individual cell types with different compensation settings. Results show mean and SD of at least four independent experiments per cell type. Statistical analysis was performed as described in Figure 3.

To investigate whether stimulation of different immune cells with minimal LPS concentrations would also result in the up-regulation of typical co-stimulatory molecules, we analysed the surface expression of CD40, CD80, CD83 and CD86 by flow cytometry (**Figure 4**). Again, THP-1 cells were the least sensitive cell type and required 2 ng/ml LPS to show minor changes in surface marker expression. Similar to our observations with cytokine secretion, moDCs required 2 ng/ml LPS to induce expression of all activation markers as well, but the changes in surface marker expression were again of much higher significance than in THP-1 cells. Whereas monocytes are highly sensitive to LPS and showed up-regulation of CD40 and CD80 in response to 0.02 ng/ml LPS, CD1c^+^ DCs seem to be even more sensitive, as they additionally expressed CD86 at these minimal LPS concentrations.

**Figure 5:**
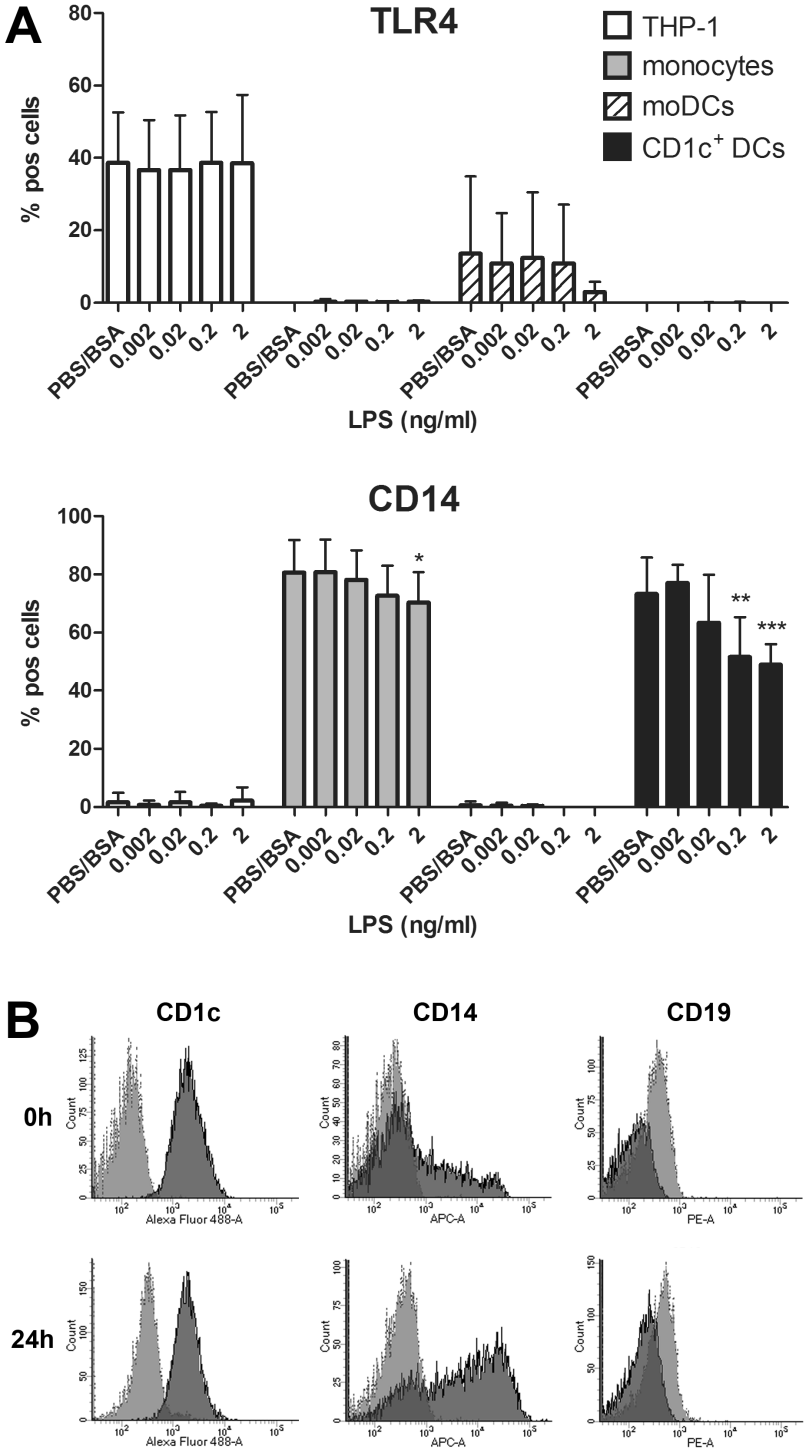
Endotoxin sensitivity correlates with CD14 expression. **A** Expression of TLR4 and CD14 on different human immune cells. Cells were plated and induced as described in Figure 3. After 24 h, cells were stained for the expression of TLR4 and CD14 and analysed by flow cytometry. Results show the percentage of fluorescence-positive cells (% pos cells) compared to the respective isotype controls. Statistical analysis was performed as described in Figure 3. **B** Analysis of CD1c, CD14 and CD19 expression on primary human CD1c^+^ DCs. 1×10^5^ CD1c^+^ DCs were stained for the expression of CD1c, CD14 or CD19 either directly after isolation (0 h), or after 24 h culture in 24-well plates containing 1 ml of DC-medium. Cells were then analysed by flow cytometry. One representative donor is shown. Light grey, isotype control; dark grey, respective surface marker staining.

Binding of LPS brings CD14, MD-2 and TLR4 in close proximity, thereby forming the LPS receptor complex [4]. Formation and activation of this complex results in docking of different adapter molecules, which induces a first wave of NF-kB activation [5], [14]. Whereas CD14 binds the carbohydrate portion of LPS and is thus mainly involved in LPS recognition [15], TLR4 acts as the main signalling receptor for LPS [16]. To analyse whether the high LPS sensitivity of monocytes and especially CD1c^+^ DCs was related to the expression of CD14 and/or TLR4, we compared the expression of both surface molecules on all four cell types. Whereas in monocytes and CD1c^+^ DCs TLR4 is hardly detectable, a major fraction of both cell types expresses CD14, which is significantly down-regulated with increasing concentrations of LPS. In contrast, THP1 cells and moDCs express TLR4 on their surface, but lack substantial CD14 expression (**Figure 5A**). Although a minor population of freshly isolated, blood-derived human CD1c^+^ DCs was previously shown to express CD14 [17], [18], the high number of CD14-expressing CD1c^+^ DCs observed in our experimental setup was rather unexpected. Because we monitored CD14 expression 24 h after cell isolation, we cultured CD1c^+^ DCs in medium for 24 h and compared CD14 expression to cells that have been freshly isolated. In line with previous studies [17], [18], only a minor fraction of CD1c^+^ DCs was found to express CD14 immediately after cell isolation, whereas CD14 expression was drastically increased after culturing the cells in DC-medium for 24 h (**Figure 5B**).

## Discussion

The present study aimed at investigating the effects of very low LPS concentrations on human immune cells. We show that CD1c^+^ dendritic cells especially can be activated by minimal amounts of LPS, equivalent to the levels of endotoxin contamination we detected in some commercially available proteins. THP-1 cells were the least sensitive cell type, which might be explained by the fact that they represent a relatively immature type in the monocyte-macrophage cell lineage that expresses low levels of CD14 [19]. As CD14-deficient monocytes are characterised by poor LPS uptake [20], low CD14 expression in THP-1 cells could result in reduced sensitivity to LPS. Although CD14^+^ monocytes have been used as precursors for the generation of moDCs, the latter have a typical DC-like morphology. moDCs express high levels of CD1a but lack CD14 [21], which may again account for the lower LPS sensitivity of these cells compared to monocytes. Interestingly, CD1c^+^ DCs are classified as myeloid DCs, the majority of which are CD14^−^ [22]. Yet, a minor fraction of these cells was previously described to express CD14 [17], [18]. In the present study we clearly show that CD1c^+^ DCs maintained in cell culture medium for 24 hours express increased levels of CD14. CD14 was shown to bind LPS at picomolar concentrations and to be critically involved in controlling endotoxin sensitivity especially to low concentrations of LPS [23], [24]. We therefore assume that the high CD14 expression on CD1c^+^ DCs observed after 24 hours of culturing significantly contributes to the enhanced sensitivity of these cells and allows for LPS-induced cytokine secretion and surface marker expression, despite the fact that TLR4 expression is rather low in those cells. However, besides CD14, other proteins as well, including LPS-binding protein, the secreted glycoprotein MD-2 and a number of adaptor proteins, contribute to LPS binding and LPS-induced signal transduction [4] and may thus be important candidates for further investigation.

In conclusion, we showed that primary human immune cells, especially CD1c^+^ DCs, are highly sensitive to LPS and can be activated by LPS concentrations as low as 0.02 ng/ml. This observation is of high significance because 0.02 ng LPS is equivalent to the amount of endotoxin impurities that may be present in 100 ng recombinant protein. Hence, the amounts of endotoxin impurities found in commercially available recombinant proteins might be sufficient to activate immune cells. Even if the LPS impurities alone do not affect those cells, it has to be considered that low LPS concentrations together with other types of stimuli (e.g. cytokines or NOD-like receptor ligands) could have synergistic effects [25]–[27] and thus produce erroneous data. To avoid endotoxin contamination that may compromise research experiments, we recommend working with proteins that have been expressed under largely endotoxin-free conditions. This includes either expression of recombinant proteins by non-bacterial expression systems like HEK293 cells, or the use of a new line of competent cells called ClearColi, an *E. coli* strain possessing a genetically modified LPS that does not induce inflammatory responses in human cells. Although other potential bacterial components (e.g. peptidoglycans or lipoproteins) may contaminate recombinant proteins, LPS remains the main concern due to its heat stability [28], binding affinity to different surfaces [29], and its ability to elicit immune responses at very low concentrations [30]. Therefore, we strongly advise using the methods mentioned in Table 1, Figure 1 and Figure 2 to ensure that all proteins used are devoid of endotoxin impurities, especially when working with immune cells that are highly sensitive to LPS.
